# Supplementary material for: Successional Shifts in Soil C:N:P stoichiometry and its environmental controls during forest restoration in Huize County, Qujing, Yunnan Province
Source: PLoS One. 2026 Aug 21;21(8):e0356261. doi: 10.1371/journal.pone.0356261 (PMC13496410; doi:10.1371/journal.pone.0356261)
Supplement: Supplementary file 2 [file pone.0356261.s004.zip › Data and Code/RDA/Pinus yunnanensis forest/RDA-2.pdf]

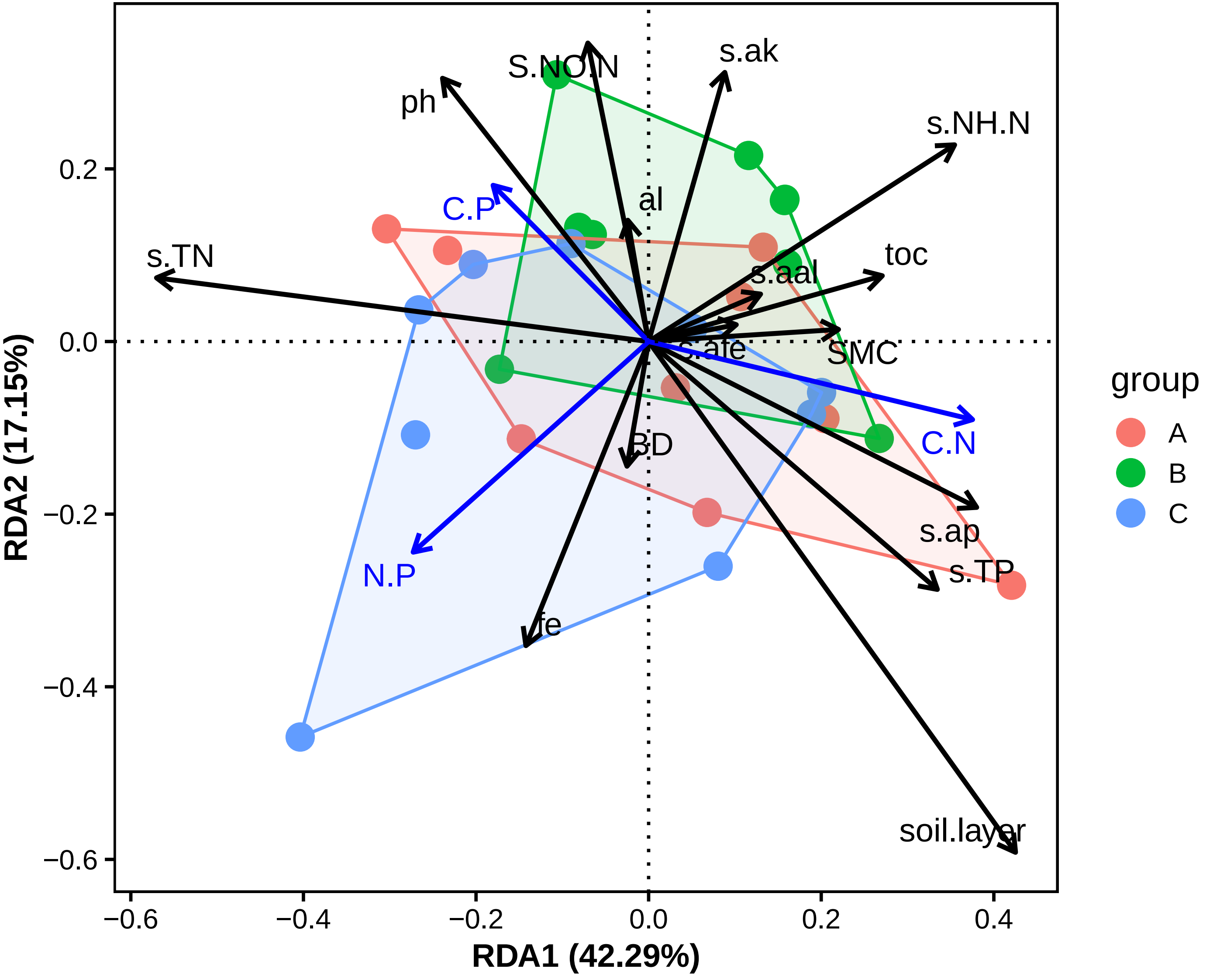

S.NO.N
s.ak
ph
s.NH.N
0.2
C.P
al
s.TN
toc
s.aal
0.0
s.afe
SMC
group
A
B
C
BD
C.N
−0.2
s.ap
N.P
s.TP
fe
−0.4
soil.layer
−0.6
−0.6
−0.4
−0.2
0.0
0.2
0.4
RDA2 (17.15%)
RDA1 (42.29%)
